# MiR338-3p expression in extracellular vesicles after severe trauma with or without traumatic brain injury

**DOI:** 10.1093/braincomms/fcaf242

**Published:** 2025-06-21

**Authors:** Jason-Alexander Hörauf, Liudmila Leppik, Birte Weber, Frank Hildebrand, Philipp Störmann, Dirk Henrich, Ingo Marzi, Cora Rebecca Schindler

**Affiliations:** Department of Trauma Surgery and Orthopedics, Goethe University Frankfurt, University Hospital, Frankfurt am Main 60590, Germany; Department of Trauma Surgery and Orthopedics, Goethe University Frankfurt, University Hospital, Frankfurt am Main 60590, Germany; Department of Trauma Surgery and Orthopedics, Goethe University Frankfurt, University Hospital, Frankfurt am Main 60590, Germany; Department of Trauma and Reconstructive Surgery, University Hospital RWTH Aachen, Aachen 52074, Germany; Department of Trauma Surgery and Orthopedics, Goethe University Frankfurt, University Hospital, Frankfurt am Main 60590, Germany; Department of Trauma Surgery and Orthopedics, Goethe University Frankfurt, University Hospital, Frankfurt am Main 60590, Germany; Department of Trauma Surgery and Orthopedics, Goethe University Frankfurt, University Hospital, Frankfurt am Main 60590, Germany; Department of Trauma Surgery and Orthopedics, Goethe University Frankfurt, University Hospital, Frankfurt am Main 60590, Germany

**Keywords:** traumatic brain injury, extracellular vesicles, biomarkers, miRNA, polytrauma

## Abstract

Polytrauma and severe traumatic brain injury (TBI) are major global health burdens associated with high morbidity and mortality, requiring accurate and early diagnosis to prevent secondary complications. In polytrauma, current protein-based biomarkers for TBI lack specificity, highlighting the need for novel approaches. Circulating microRNAs, especially those encapsulated in extracellular vesicles, represent a promising alternative due to their stability and tissue-specific signatures. This study investigates compartment-specific expression of candidate microRNAs in plasma, total extracellular vesicles and neuro-derived extracellular vesicles following trauma. In this retrospective analysis, blood samples were prospectively collected from 20 trauma patients (Injury Severity Score ≥ 16; *n* = 10 polytrauma without TBI and *n* = 10 with isolated TBI) and 10 healthy controls at two time points (≤3 and 48 h post-injury). Extracellular vesicles were isolated from plasma, and neuron-derived extracellular vesicles were enriched using L1CAM-coated magnetic beads. Expression of five microRNAs (miR-21-5p, miR-142-3p, miR-191-5p, miR-192-3p and miR-338-3p) was quantified via real-time polymerase chain reaction in plasma and in (neuron-derived) extracellular vesicles. miR-21-5p was significantly elevated in plasma shortly after polytrauma and correlated with injury severity, leukocytosis and prolonged stay on intensive care unit. In TBI patients, higher injury severity scores correlate with worse outcomes, while intracranial pressure was linked to improved recovery. miR-192-3p was predominantly enriched in neuron-derived extracellular vesicles. After polytrauma, its levels in neuron-derived extracellular vesicles were further elevated but declined after 48 h. In contrast, after TBI, miR-192-3p levels in neuron-derived extracellular vesicles initially decreased compared with healthy controls and increased again at 48 h. miR-338-3p exhibited time- and injury-dependent expression patterns. After polytrauma, it was significantly elevated in plasma and extracellular vesicles within the first hours and was associated with inflammation, organ failure and prolonged ventilation. After TBI, miR-338-3p showed a marked increase in neuron-derived extracellular vesicles at 48 h. Compartment-specific microRNA profiling reveals distinct molecular signatures after polytrauma and TBI. Increased miR-21-5p levels after polytrauma were associated with inflammation and worse clinical condition. miR-192-3p was enriched in neuron-derived extracellular vesicles in healthy controls but decreased early after TBI. miR-338-3p showed a context-dependent pattern: increased rapidly in plasma and extracellular vesicles after polytrauma but showed a delayed, TBI-specific upregulation in neuron-derived extracellular vesicles at 48 h, suggesting a potential role in CNS-specific injury or repair processes.

## Teaser

Schindler *et al*. report distinct time- and injury-dependent microRNA signatures after (poly)trauma. miR-338-3p shows delayed upregulation in neuron-derived extracellular vesicles following traumatic brain injury, indicating its role in secondary CNS responses. Findings highlight the diagnostic potential of compartment-specific microRNA profiling in trauma care.

## Graphical Abstract

**Graphical Abstract:**
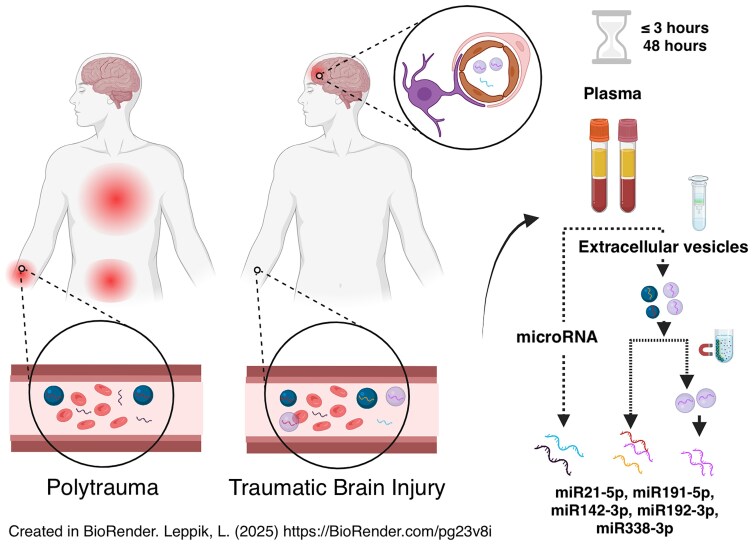

## Introduction

Polytrauma (PT) and traumatic brain injury (TBI), caused by various accident mechanisms such as falls, traffic accidents, sports or violence, are among the most common causes of severe disability and death worldwide.^1^ Understanding the pathophysiology of the post-traumatic response is essential for developing more effective diagnostic and treatment strategies to minimize the consequences of severe injury. Over the years, medical research into immunomodulatory processes following PT has made significant advances. However, TBI remains the leading cause of death in PT, alongside haemorrhagic shock.^2,3^

The pathophysiology of TBI is a complex process initiated by direct mechanical forces and sustained by subsequent cellular and molecular responses. The external impact causes deformation of brain tissue, potentially leading to the rupture of blood vessels, axons and neurons. This primary injury triggers a cascade of inflammatory processes and neurochemical reactions, resulting in the release of neurotransmitters, cytokines and reactive oxygen species.^4^ These processes, combined with the brain's attempts at repair and regeneration, can lead to permanent neuronal alterations and cell death, ultimately causing long-term neurological deficits. The clinical manifestations of TBI are diverse, ranging from transient loss of consciousness to persistent cognitive, emotional and motor impairments.^5-7^ Accurate diagnosis and classification of TBI remain challenging due to its complex nature and variability of clinical outcomes among individuals.^8^ Clinical assessment and cranial computed tomography (CT) continue to serve as the gold standard for diagnosis.^9^ Biomarkers—molecules or substances that cross the compromised blood–brain barrier (BBB) after injury and can be detected in peripheral blood—represent a promising diagnostic adjunct, as they may reflect injury severity and progression.^10^ Among the most extensively studied are neuro- and glia-specific proteins such as the calcium-binding protein S100B, glial fibrillary acidic protein and neuron-specific enolase, which are elevated in the bloodstream following TBI.^11^ However, in the context of PT, these protein-based biomarkers often lack specificity due to the involvement of multiple organs and the systemic release of these molecules.^12^

In this context, microRNAs (miRNAs) have emerged as promising candidates for both the diagnosis and prognosis of TBI and may even serve as therapeutic targets.^13^ miRNAs are small, non-coding RNA molecules that play a critical role in post-transcriptional gene regulation and can influence a wide range of biological processes. The clinical utility of circulating miRNAs as early diagnostic and prognostic biomarkers has also been demonstrated in cardiovascular diseases such as coronary artery disease and acute coronary syndrome. For example, miRNAs such as miR-137 and miR-106b-5p have been shown to detect myocardial ischaemia in patients with unstable angina and to stratify disease severity, with minimal expression in healthy individuals.^14^ Likewise, miR-223-5p was identified as a diagnostic and prognostic marker for acute coronary syndrome, correlating with established clinical parameters such as troponin I and predicting the occurrence of major adverse cardiovascular events following percutaneous coronary intervention (Zhang *et al*. 2024).^15^ These findings highlight the translational potential of miRNAs across a spectrum of acute injury conditions and support their further investigation in trauma settings, where early diagnosis and individualized risk stratification remain critical challenges. Our preliminary work, along with findings from other research groups, has highlighted the potential of miRNAs as blood-based biomarkers in TBI.^16^

miRNAs can cross the BBB either freely due to their small size or enclosed within extracellular vesicles (EVs). EVs are small, membrane-bound particles released from cells that carry proteins, RNAs and DNA, whose surface markers and molecular cargo can reflect their cellular origin.^17^ In the context of trauma, EVs may provide crucial insights into the extent of injury and underlying pathophysiological mechanisms.^18^ Both the vesicles themselves and their contents are being explored as novel biomarker platforms to enhance the diagnosis, prognosis and management of TBI patients.^19,20^

In this study, we focused on selected miRNAs that have previously demonstrated time- and injury-dependent changes in plasma (miR21-5p, miR338-3p and miR142-3p), as well as miRNAs identified as differentially expressed in prior deep sequencing analyses of TBI and PT patients (miR191-5p and miR192-3p). miR-21-5p has consistently been reported as a biomarker of severe TBI, showing upregulation in serum and cerebrospinal fluid and correlating with injury severity and clinical outcome.^13,21^ Beyond its diagnostic potential, it is also involved in neuroinflammatory signalling and glial activation.^22^ miR-338-3p has been linked to neurodegenerative processes, particularly in amyotrophic lateral sclerosis, where it is elevated in spinal cord, cerebrospinal fluid and blood, and contributes to metabolic dysfunction.^23,24^ Given its role in regulating neuronal energy metabolism, miR-338-3p may also play a role in secondary injury mechanisms following TBI. miR-142-3p upregulation was seen in microglia, macrophages and lymphocytes in perilesional brain regions, and its overexpression in experimental TBI models has been shown to enhance astrocyte reactivity, suggesting a role in amplifying neuroinflammation and contributing to secondary brain injury.^25,26^ Additionally, changes in plasma levels of miR-142-3p have been linked to cognitive impairment and the development of post-concussive symptoms, suggesting its potential as a prognostic biomarker for neurocognitive recovery following TBI.^27^ miR-191-5p showed disease-specific expression patterns in cerebrospinal fluid and plasma of patients with Alzheimer's disease and disorders of consciousness after TBI.^28,29^ By reducing tau phosphorylation, amyloid-β production and neuronal apoptosis, it may serve as a neuroprotective biomarker.^30^ Finally, miR-192-3p has been identified as a circulating marker of poor functional outcome in patients with acute ischaemic stroke undergoing thrombolysis.^31^ It is also elevated in amyotrophic lateral sclerosis and may reflect broader neuronal stress responses and degeneration.^32^

Plasma miRNAs reflect a heterogeneous mix of systemic signals and are easily accessible but often lack tissue specificity and stability.^33^ In contrast, EV-associated miRNAs—especially those in neuron-derived EVs—are protected from degradation and may more specifically reflect CNS cellular activity.^34^ Notably, neuron-derived EVs have revealed stage-specific changes in protein biomarkers across acute and chronic TBI, suggesting their potential as longitudinal indicators of injury progression and cognitive decline.^35,36^ miR-21-5p in neuronal exosomes has been shown to promote microglial M1 polarization and neuroinflammation,^37^ while also exerting neuroprotective effects by limiting autophagy-mediated neuronal damage,^23^ underscoring its context- and cell-type-specific functions. Furthermore, neuron-derived EVs have been demonstrated to actively modulate the CNS microenvironment, supporting remyelination, suppressing glial activation and promoting neuronal regeneration in models of spinal cord injury.^38^ These findings reinforce the notion that exosomal and neuron-derived miRNAs are not merely byproducts of injury but may play active roles in post-traumatic signalling and recovery. Thus, the identification and validation of such biomarkers hold the potential to significantly improve the diagnostic accuracy and enable more personalized treatment strategies.

Here, we aim to isolate EVs from the blood of trauma patients and analyse their miRNA content, with the goal of identifying specific signatures associated with TBI. These findings may ultimately contribute to enhanced diagnostic tools, targeted therapeutic approaches and improved outcomes for individuals affected by TBI.

## Materials and methods

### Patients and compliance with ethical requirements

This study has been conducted after approval by the Institutional Review Board of the University Hospital of the Goethe University Frankfurt (89/19). Written informed consent was obtained for enrolled patients and volunteers or their legally authorized representatives.

The study was carried out at the Hospital of Goethe University Frankfurt, Germany, following approval by the Institutional Review Board (89/19). Written informed consent was obtained from all participating patients or their legal representatives. We performed a retrospective analysis of prospectively collected data in accordance with the Declaration of Helsinki and the Strengthening the Reporting of Observational Studies in Epidemiology (STROBE) guidelines.^39^ We included *n* = 20 severely injured trauma patients [Injury Severity Score (ISS) ≥ 16], aged ≥18 years, categorized into two groups: multiple trauma patients without TBI [(PT), AIS_head_ = 0] and patients with severe isolated TBI [(TBI), AIS_head_ ≥ 4, with all other AIS ≤ 1]. Healthy volunteers (Ctrl) served as the control group.

Injury severity was assessed using the ISS, which is derived from the Abbreviated Injury Scale (AIS). The AIS assigns a severity score ranging from 1 (minor) to 6 (unsurvivable) across six body regions: head, face, thorax, abdomen, extremities (including pelvis) and external injuries.^40,41^ To better reflect the overall trauma burden, the New ISS (NISS) was also calculated.^42^

#### Identification of EVs and miRNAs

Blood samples were collected from patients admitted to the emergency department of our Level 1 Trauma Center at the University Hospital of Goethe University Frankfurt within 3 h [time of emergency room (ER) admission] and again at 48 h post-injury. Blood was drawn according to standard hospital protocols into pre-chilled ethylenediaminetetraacetic acid tubes. Samples were centrifuged at 3000 × *g* for 15 min at 4°C, and the resulting plasma supernatant was stored at −80°C until further analysis. Plasma concentrations of Interleukin (IL)-6 and IL-10 (normal < 7.0 pg/mL) were measured using IL-6/IL-10 Eli-pair Enzyme-linked Immunosorbent Assay. White blood cell (WBC) counts (normal range: 3.92–9.81 WBC/nL) were obtained from routine clinical laboratory results documented in the patient records. EVs were isolated using size exclusion chromatography. To enrich for neuro-specific EVs (nEVs), magnetic beads were conjugated with a biotinylated L1CAM antibody. EV identity was confirmed by Western blot analysis for CD63, nanoparticle tracking analysis and transmission electron microscopy, as previously described.^43,44^ EVs were incubated with the L1CAM-coated magnetic beads, and bead-bound EVs were isolated using a magnetic separator. These were directly processed for miRNA extraction (RNeasy Kit, Qiagen), followed by cDNA synthesis. Quantitative real-time polymerase chain reaction was performed under the following cycling conditions: 1 cycle at 95°C for 3 min, followed by 40 cycles at 95°C for 10 s and 56°C for 50 s, ending with melting curve analysis. To normalize for variation in RNA extraction and reverse transcription efficiency, the exogenous spike-in control *cel*-miR-39 was measured. All procedures were performed in accordance to the manufacturer's instructions. A detailed description of the EV isolation and miRNA extraction protocol, including the size exclusion chromatography procedure and associated quality control measures, is provided in the Supplementary material ‘Patients and Methods’.

#### Statistical analysis

For the relative quantification of target miRNAs, the delta Ct (2^−ΔCT^) and delta-delta Ct (2^−ΔΔCT^) methods were applied according to Schmittgen and Livak and as previously described.^45^ The absence of a standardized housekeeping gene for miRNA normalization remains a major methodological challenge. Therefore, the exogenous spike-in control *cel*-miR-39 was used for normalization. Continuous variables with normal distribution are presented as mean ± standard deviation, while categorical variables and non-normally distributed continuous variables are reported as median with interquartile range (IQR). *P*-values for categorical variables were calculated using *χ*^2^ test, while those for continuous variables were derived from the ANOVA [*F* (*d*1; *d*2)] or Kruskal–Wallis. *Post hoc* analyses were adjusted for multiple comparisons using the Bonferroni correction. Pearson's (parametric) and Spearman's (non-parametric) correlation coefficients were used to assess the relationships between miRNA expression levels and injury characteristics. A *P*-value < 0.05 was considered statistically significant.

All statistical analyses were performed using the Statistical Package for Social Sciences (SPSS for Mac©), version 28 (SPSS Inc., Chicago, IL, USA). Graphs were created using GraphPad Prism 7 for Mac© (GraphPad Software Inc., San Diego, CA, USA).

## Results

This study includes *n* = 20 trauma patients with an ISS ≥ 16 and *n* = 10 healthy volunteers (Table 1). Among the patients, *n* = 10 had sustained severe isolated TBI, with a median age of 49 years (IQR 26–73) and a median NISS of 36 points (IQR 32–49). The remaining *n* = 10 patients were multiply injured without TBI (PT), with a median age of 41 years (IQR 29–54) and median NISS of 40 points (IQR 33–50). Systemic and EV-associated levels of miR21-5p, miR191-5p, miR338-3p, miR142-3p and miR191-3p were quantified using real-time polymerase chain reaction and compared across the study groups.

**Table 1:** Demographic and clinical characteristics

Patients	TBI (*n* = 10)	PT (*n* = 10)	Healthy (*n* = 10)
Age (y, median; IQR)	49 (26–73)	41 (29–54)	40 (37–54)
Sex (m:f)	8:2	9:1	6:4

Injury scores (pts)					
	Median	IQR	Median	IQR	
ISS	29	26–33	34	29–40	*P* = 0.057
NISS	36	32–49	40	33–50	*P* = 0.838
AIS_head_	5	4–5	0	0	
AIS_face_	0	0–3	0	0	
AIS_thorax_	0	0–1	3	3–5	
AIS_abdomen_	0	0	2.5	2–4	
AIS_extremity_	0	0	3.5	3–5	

(N)ISS, (New) Injury Severity Score; AIS, Abbreviated Injury Scale; IQR, interquartile range; PT, polytrauma; TBI, traumatic brain injury.

### miR192-3p is predominantly content of nEV in healthy human

**Figure 1:**
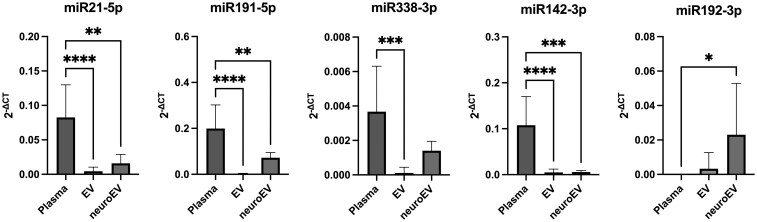
**miRNA concentrations in plasma, EVs and nEVs of healthy controls.** miR21-5p, miR423-3p, miR142-3p, miR191-5p and miR338-3p are predominantly detected in the plasma of healthy individuals (*n* = 10). In contrast, miR192-3p is significantly more [*F*(2,21) = 4.42; *P* = 0.025] abundant in nEVs. 2^−ΔCT^ = delta CT Method. ANOVA: F (DFn, DFd). *P < 0.05, **P < 0.01, ***P < 0.001, and ****P < 0.0001. (neuro)EV, (neuron-specific) extracellular vesicles.

Figure 1 illustrates the 2^−ΔCT^ values of the investigated miRNAs in plasma, total EVs and nEVs from healthy controls. nEVs were enriched using the surface marker L1CAM. A comparison between plasma and EV-derived miRNA content showed that miR21-5p, miR191-5p, miR338-3p and miR142-3p were significantly more abundant in plasma than in total EVs (*P* < 0.001; Fig. 1). However, their levels appeared elevated in nEVs compared with total EVs (Fig. 1; Table 2). Notably, miR192-3p is predominantly detected in nEVs [*F*(2,21) = 4.42; *P* = 0.025; Fig. 1].

**Table 2:** Delta CT values of healthy controls, after PT and TBI in plasma, EV and nEV

2^−ΔCT^	Ctrl	PT		TBI	
		ER	48 h	ER	48 h
Plasma					
miR21-5p	0.083 ± 0.047	0.701 ± 0.450***	0.105 ± 0.049	0.161 ± 0.113	0.151 ± 0.123
miR191-5p	0.199 ± 0.103	0.523 ± 0.921*	0.070 ± 0.019	0.312 ± 0.266	0.004 ± 0.004
miR338-3p	0.004 ± 0.003	0.017 ± 0.018*	0.002 ± 0.001^##^	0.013 ± 0.014	0.005 ± 0.006
miR142-3p	0.108 ± 0.062	0.170 ± 0.240	0.030 ± 0.016	0.132 ± 0.178	0.063 ± 0.059
miR192-3p	0.000^#^	0.000^###^	0.000^#^	0.0002 ± 0.0004	0.0001 ± 0.0004^#^
EV					
miR21-5p	0.005 ± 0.006^###^	0.008 ± 0.012^###^	0.001 ± 0.001	0.005 ± 0.007^###^	0.006 ± 0.006^##^
miR191-5p	0.002 ± 0.002^###^	0.008 ± 0.009^#^	0.008 ± 0.006	0.007 ± 0.006^##^	0.004 ± 0.004
miR338-3p	0.0001 ± 0.0003^###^	0.001 ± 0.002*	0.000^###^	0.0005 ± 0.0005^#^	0.0001 ± 0.0004
miR142-3p	0.005 ± 0.007^###^	0.010 ± 0.012^#^	0.003 ± 0.003^##^	0.006 ± 0.004^#^	0.008 ± 0.008
miR192-3p	0.003 ± 0.009	0.003 ± 0.0005^###^	0.0001 ± 0.003^#^	0.0006 ± 0.001	0.0004 ± 0.0005^#^
nEV					
miR21-5p	0.016 ± 0.013^##^	0.066 ± 0.071^###^	0.039 ± 0.034	0.021 ± 0.019^##^	0.032 ± 0.022^#^
miR191-5p	0.072 ± 0.023^##^	0.212 ± 0.009*	0.102 ± 0.084	0.143 ± 0.064	0.164 ± 0.087
miR338-3p	0.001 ± 0.0005	0.013 ± 0.010	0.012 ± 0.010	0.010 ± 0.012**	0.008 ± 0.013***
miR142-3p	0.006 ± 0.004^##^	0.119 ± 0.127	0.045 ± 0.018	0.014 ± 0.007	0.028 ± 0.034
miR192-3p	0.023 ± 0.030	0.062 ± 0.070	0.021 ± 0.029	0.008 ± 0.014	0.013 ± 0.016

Ctrl, control; ER, emergency room; (n)EV, (neuro-specific) extracellular vesicles; PT, polytrauma; TBI, traumatic brain injury.

ANOVA test (*n* = 10 per group): * = comparing Ctrl; ^#^ = miRNA level between plasma, EV and nEV.

^*/#^ *P* < 0.05.

^**/##^ *P* < 0.01.

^***/###^ *P* < 0.001.

**Figure 2:**
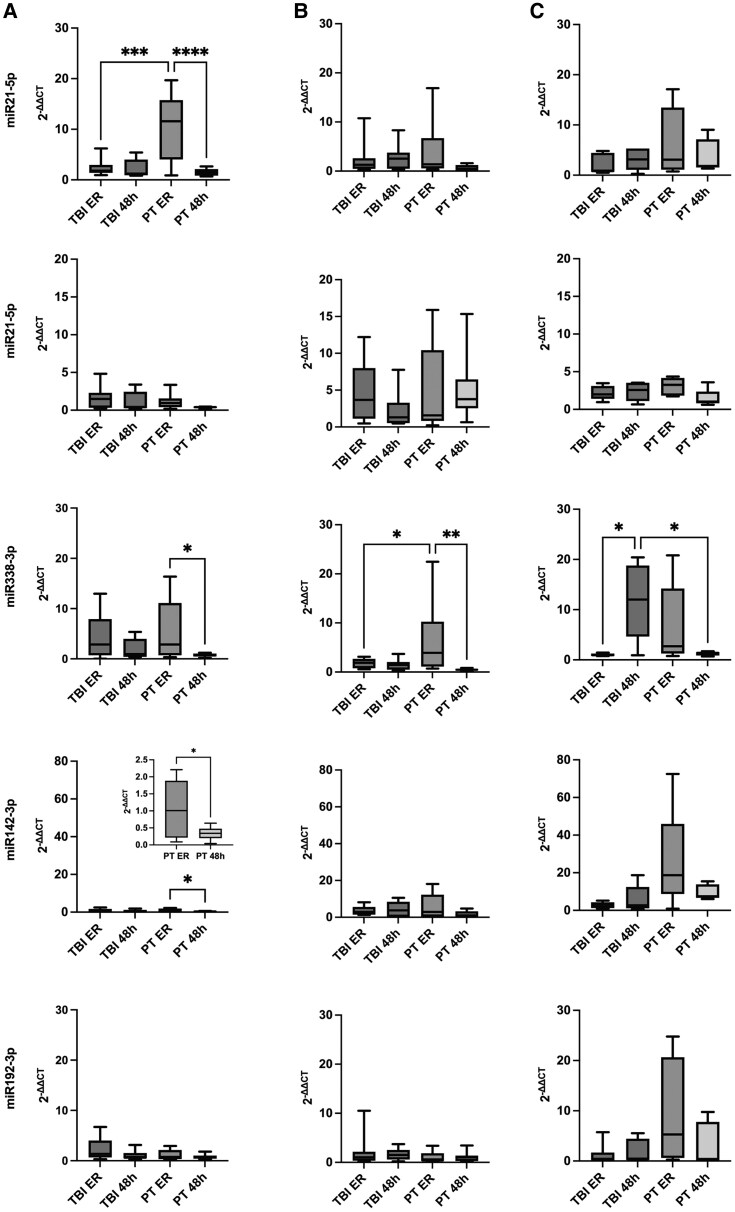
**miRNA content in plasma, EVs and nEVs.** Following PT without TBI (*n* = 10), plasma levels of miR21-5p were markedly elevated upon admission [ER; *F*(3,31) = 7.54; *P* < 0.001], with a time-dependent decrease observed after 48 h (**A**). miR191-5p levels were increased in total EVs after both TBI (*n* = 10) and PT (**B**). miR338-3p was significantly upregulated in plasma and EVs [*F*(3,30) = 4625; *P* = 0.009] during the early post-PT phase (**A**, **B**), whereas a distinct increase was observed in nEVs 48 h after isolated TBI [*F*(3,17) = 4.02; *P* = 0.025] (**C**). Both miR142-3p and miR192-3p showed elevated levels in nEVs up to 48 h after trauma (**C**). 2^−ΔCT^ = delta CT Method. ANOVA: *F* (DFn, DFd). *P < 0.05, **P < 0.01, ***P < 0.001, and ****P < 0.0001. h, hours; ER, emergency room; (neuro)EV, (neuro-specific) extracellular vesicles; PT, polytrauma; TBI, traumatic brain injury.

The 2^−ΔCT^ values of healthy controls, PT and TBI patients in plasma, EVs and nEVs are presented in Table 2. Figure 2 illustrates the 2^−ΔΔCT^ values, showing fold changes in miRNA expression relative to the healthy control group.

In PT patients without TBI, plasma levels of miR21-5p were markedly elevated at the time of admission to the ER [*F*(3,31) = 7.54; *P* < 0.001]. A time-dependent decline in miR-21-5p was observed at 48 h post-injury. In contrast, miR21-5p levels in nEVs remained mildly elevated at 48 h.

The 2^−ΔΔCT^ values of miR191-5p were elevated in EVs following both TBI and PT. However, no statistically significant differences were observed between the groups or time points (Fig. 2).

In the early phase following PT, miR338-3p levels were increased in both plasma and EVs compared with 48 h and to TBI. In contrast, miR338-3p in nEVs showed a distinct pattern: it was significantly upregulated 48 h after isolated TBI, with a 10-fold increase in 2^−ΔΔCT^ values compared with PT and the ER time point [*F*(3,17) = 2.64; *P* = 0.025; Fig. 2].

Plasma levels of miR142-3p showed a modest increase at ER admission after PT. However, nEV-associated miR142-3p appeared to be markedly elevated in the early phase after PT compared with healthy controls (Fig. 2).

Although miR192-3p was predominantly found in nEVs across all groups, levels were further increased after PT and subsequently declined at 48 h. Following TBI, nEV-associated miR192-3p initially decreased compared with healthy controls, but levels rose again at the 48-h time point (Table 2).

**Figure 3:**
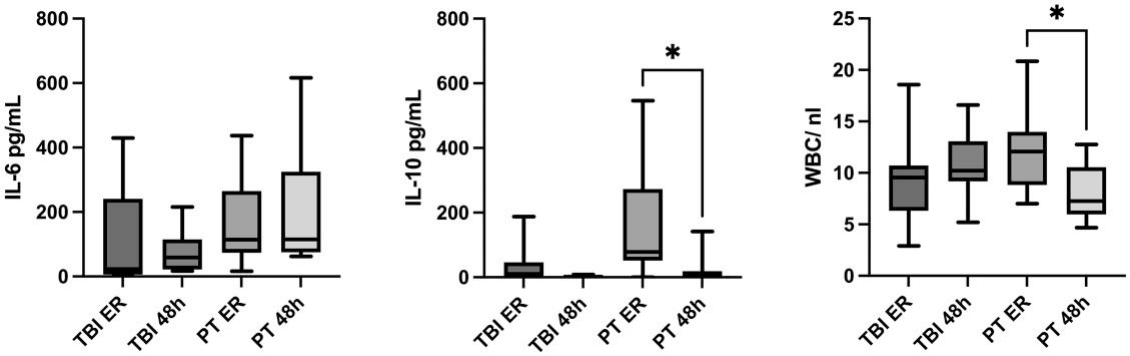
**Plasma concentrations of clinical inflammation markers WBC, IL-6 and IL-10. WBC (norm 3.92–9.81 WBC/nL), IL-6 (norm < 7.0 pg/mL) and IL-10 (norm < 7.0 pg/mL) show increased release within the first hours after trauma.** WBC (*P* = 0.016) and IL-10 (*P* = 0.036) concentrations drop again 48 h after PT (*n* = 10) and IL-6 continues to rise. Foryt-eight hours after TBI (*n* = 10), IL-6 and IL-10 level decrease, while WBC level remain elevated. Kruskal–Wallis test: *P < 0.05, **P < 0.01, ***P < 0.001, and ****P < 0.0001. h, hours; ER, emergency room; PT, polytrauma; TBI, traumatic brain injury; (neuro)EV, (neuro-specific) extracellular vesicles; IL, interleukin; WBC, white blood cell.

Figure 3 shows plasma concentrations of clinical inflammation markers WBC, IL-6 and IL-10. All IL-6, IL-10 and WBC showed increased levels within the first hours after trauma. Following PT, WBC and IL-10 levels declined after 48 h, whereas IL-6 remained elevated. In contrast, after TBI, IL-6 and IL-10 concentrations decreased by 48 h, while WBC levels stayed elevated. To explore potential associations between inflammatory responses and miRNA expression patterns, we performed correlation analyses.

Within the first hours after PT, plasma levels of miR21-5p and miR338-3p showed a strong positive correlation (rho 0.867, *P* = 0.002), and miR142-3p also correlated positively with both (rho 0.750, *P* = 0.020). At this time point, WBC counts were positively correlated with miR21-5p (rho 0.738, *P* = 0.023), miR338-3p (rho 0,715, *P* = 0.020) and miR142-3p (rho 0.809, *P* = 0.005). At 48 h post-injury, the correlation pattern between WBC and these miRNAs shifted towards a negative trend, although these associations were not statistically significant. In total EVs, miR191-3p correlated positively with IL-6 (rho 0.840, *P* = 0.009), and EV-associated miR142-3p correlated with WBC levels (0.787, *P* = 0.012). In nEVs, strong positive correlations were observed between IL-10 and miR338-3p (rho 0.941, *P* = 0.017) as well as miR192-3p (rho 0.973, *P* = 0.027).

In patients with TBI, plasma levels of miR21-5p, miR192-5p, miR142-3p and miR338-3p were positively correlated at the time of admission. However, no significant correlations with inflammatory markers were observed in this group.

To further evaluate the diagnostic and prognostic potential of the investigated miRNAs, correlation analyses were performed with clinical outcome parameters (see Table 3).

**Table 3:** Clinical and outcome parameter stratified by injury pattern

	TBI (*n* = 10)	PT (*n* = 10)
Intensive care		
GCS (median IQR)	3 (3–6)	15 (10–15)
ICU (%)	90	100
ICU (d, median IQR)	8 (2–21)	13 (8–21)
ETI (%)	75	25
ETI (d, median IQR)	2 (1–8)	3 (2–5)
MOF (%)	11.1	-
Pulmo	33.3	10
Coagulation	11.1	20
Heart	22.2	30
CNS	66.7	10
Kidney	-	10
Outcome		
RISC II (median IQR)	8 (5–76)	3 (2–6)
GOS (median IQR)	3 (1–4)	4 (4–5)
Fit (GOS 5)	11.1%	40%
Moderately disabled (GOS 4)	33.3%	40%
Severely disabled (GOS 3)	11.1%	20%
Vegetative (GOS 2)	11.1%	-
Dead (GOS 1)	33.3%	-
Palliative (%)	20%	-

CNS, central nervous system; d, days; ETI, endotracheal intubation; GCS, Glasgow Coma Scale; GOS, Glasgow Outcome Scale; ICU, intensive care unit; IQR, Interquartile Range; MOF, multiple organ failure; PT, polytrauma; RISC, Revised Injury Severity Classification; TBI, traumatic brain injury.

In PT patients, a higher ISS was significantly associated with poorer outcomes (rho −0.758, *P* = 0.011). The severity of thoracic injury correlated positively with both the duration of mechanical ventilation (rho 0.811, *P* = 0.008) and length of stay in the intensive care unit (ICU) (rho 0.769, *P* = 0.015).

In plasma of PT patients collected upon admission, miR21-5p levels correlated positively with Revised Injury Severity Classification (RISC) II (rho 0.857, *P* = 0.014), ICU stay (rho 0.862, *P* = 0.003) and duration of ventilation (rho 0.878, *P* = 0.004). At admission, both plasma miR191-5p (rho 0.828, *P* = 0.006) and EV-associated miR191-5p (rho 0.786, *P* = 0.007) correlated positively with AIS_Thorax_. After 48 h, plasma miR191-showed a significant negative correlation with RISC II (rho −0.829, *P* = 0.021) in PT patients. Additionally, plasma miR338-3p was positively associated with AIS_Thorax_ (rho 0.759, *P* = 0.018) and negatively with RISC II (rho −0.821, *P* = 0.023) in PT patients. Neuro-EV-associated miR142-3p showed a negative correlation with patient age of PT patients 48 h post-injury (rho −0.900, *P* = 0.037). At admission, EV miR192-3p levels of PT patients were positively correlated with AIS_Thorax_ (rho 0.786, *P* = 0.007) and NISS (rho 0.675, *P* = 0.032). At this time point, nEV miR192-3p was positively associated with ICU length of stay (rho 0.900, *P* = 0.037).

In patients with TBI, NISS was negatively correlated with Glasgow Coma Scale (GCS) scores (rho −0.735, *P* = 0.015), while ISS was positively associated with RISC II (rho 0.809, *P* = 0.005). The presence of intracranial pressure monitoring correlated with better outcomes (rho 0.896, *P* = 0.001). In contrast, brain organ failure was positively associated with the need for intubation (rho 0.784, *P* = 0.007). The nEV miR338-3p was positively correlated with NISS 48 h post-TBI (rho 0.900, *P* = 0.037).

## Discussion

TBI is a major cause of neurological damage or death, with secondary inflammatory responses playing a crucial role in its progression.^1,46^ The molecular composition and regulatory function of EVs in both the CNS and systemic circulation change significantly, enabling cell-to-cell communication through the transfer of bioactive molecules such as miRNAs.^27,47^ While research on circulating miRNAs in TBI and PT is still emerging, our data offer new insights into their compartment-specific dynamics, particularly in plasma, EVs and nEVs.

### miR-21-5p: early systemic inflammatory marker after PT

In our cohort, miR21-5p levels were markedly increased in plasma during the early phase after PT without TBI (plasma > EV > nEV), followed by a decline after 48 h. Plasma levels were up to 10-fold higher than in TBI-only patients and correlated with markers of systemic inflammation (leukocytosis) and clinical severity (ICU admission, ventilation time and RISC II score). This early increase suggests its involvement in the systemic immune response and cellular stress regulation. miR21-5p is a regulatory miRNA involved in inflammation, apoptosis and cell proliferation, modulating both pro- and anti-inflammatory processes.^48^ Although miR21-5p has been associated with cell survival through anti-apoptotic and regenerative mechanisms via inhibition of phosphatase and tensin homologue and Programmed Cell Death Protein 4, its overexpression has also been linked to various cancer entities such as glioma, breast cancer and colorectal cancer.^49^ Moreover, miR21-5p upregulation appears to exert a protective function by modulating the inflammatory response and inhibiting apoptotic processes.^49^ Its non-specificity limits its use as a diagnostic marker, but its dynamic regulation still provides insight into trauma-associated pathophysiology. Weber *et al*.^50^ reported that declining EV miR-21-5p levels were associated with complications such as sepsis after PT. In this study, we observed a very early increase of miR21-5p in plasma following severe PT, which was associated with WBC release, suggesting a role in the early systemic inflammatory response. Given previous evidence linking miR-21-5p to anti-apoptotic and cell survival pathways, it is conceivable that this early peak reflects a protective mechanism during the acute stress phase. By 48 h, plasma levels decreased, possibly due to consumption, regulatory feedback or the influence of counter-regulatory pathways that become more dominant later in the inflammatory cascade. Interestingly, we observed no correlation between miR-21-5p and IL-6 or IL-10, which may reflect differing temporal expression profiles—particularly given that IL-6 peaks 24–48 h post-injury.^51^ Notably, nEV-associated miR21-5p levels remained elevated 3-fold at 48 h, which may indicate a delayed CNS-derived release, potentially due to sustained systemic activation mechanism and gradual translocation of nEVs across the BBB. We found significantly similar expression patterns and correlations for miR21-5p, miR338-3p and miR142-3p, suggesting that all these miRNAs are involved in inflammatory processes.

Although higher miR-21-5p levels have been associated with reduced neuronal and improved outcomes,^52^ we observed no significant changes in plasma and EV levels after isolated TBI. This may be due to a primarily local increase in miR21-5p within the brain, which is not reflected systematically, possibly due to the intact or partially preserved BBB during the early phase. Alternatively, a delayed systemic release may occur at later stages. Importantly, excessive miR21-5p expression in the brain may also have detrimental effects by promoting reactive gliosis and maladaptive processes.^22^ TBI is further known to induce immunosuppression, termed CNS injury-induced immunodepression. Excessive sympathetic activation and elevated stress hormones such as cortisol and catecholamines suppress immune cell function (including T cells, macrophages and dendritic cells), increasing susceptibility to infection and impairing recovery.^53^ Moreover, TBI-related BBB disruption can trigger a local inflammatory response that further suppresses systemic immunity, creating a vicious circle. Taken together, the unchanged miR21-5p levels after isolated TBI may result from the complex balance of pro- and anti-inflammatory signals, limited peripheral release or immunoregulatory mechanisms that mask measurable changes.

### miR-142-3p and miR-191-5p: indicators of neuro-inflammatory activation after PT

The concentration of miR142-3p (Fig. 2A) increases significantly in the first hours after PT. miR-142-3p showed the highest relative (to control) expression (2^−ΔΔCT^) in nEVs during the first 6 h after PT, suggesting a selective enrichment in CNS-associated compartments. This compartment-specific pattern indicates that miR-142-3p may be actively released from neuronal or glial sources in response to systemic injury, even in the absence of direct brain trauma.

Several studies have linked miR-142-3p to neuroinflammatory and synaptotoxic processes. Elevated levels were found in cerebrospinal fluid and brain tissue of patients with multiple sclerosis and in corresponding animal models, where miR-142-3p contributed to IL-1β-mediated glutamatergic synaptopathy and excitotoxic damage.^54^ Functionally, miR-142-3p downregulates the astroglial glutamate-aspartate transporter, enhancing synaptic excitability and neurodegeneration in inflammatory CNS conditions. Inhibition of miR-142-3p rescued synaptic structure and function in experimental autoimmune encephalomyelitis, further supporting its role as a mediator of neurotoxicity.^54^ Moreover, miR-142-3p has been shown to regulate key neuroplasticity pathways. In activated microglia, it suppresses Ca2+/calmodulin-dependent kinase 2a and downstream cyclic AMP-responsive element-binding protein—brain-derived neurotrophic factor signalling, which are critical for synaptic plasticity, learning and memory.^55^ In TBI, increased miR-142-3p expression has been reported in the hippocampus, potentially linking it to injury-induced cognitive dysfunction and neuronal vulnerability.^56^ Interestingly, contrasting data from Parkinson's disease models indicate that overexpression of miR-142-3p can attenuate neuronal damage and restoring autophagy balance,^57^ highlighting its context-dependent function as either neurotoxic or neuroprotective.

miR191-5p levels increased in EVs after trauma but without significant differences between trauma groups or time points. The miR-191 family has been implicated in a variety of diseases, including several cancers, Alzheimer's disease, multiple sclerosis and Type 2 diabetes, and is known to regulate key cellular processes such as proliferation, apoptosis and inflammatory signalling.^58^ While its elevation may indicate a general involvement in stress or injury responses, its broad expression profile across tissues limits its specificity and complicates clinical interpretation. Importantly, miR-191-5p has been shown to directly repress brain-derived neurotrophic factor, thereby interfering with neuronal differentiation, survival and synaptic plasticity.^59^ This is of particular relevance in the context of trauma and neurorepair, as brain-derived neurotrophic factor plays a central role in neuronal recovery following injury.^60^ Thus, elevated miR-191-5p in EVs could reflect an indirect regulatory mechanism contributing to impaired neuroplasticity or delayed regeneration post-trauma. In a recent longitudinal multiple sclerosis study, serum levels of miR-191-5p correlated positively with clinical disability and disease progression, and temporal fluctuations were associated with changes in MRI activity and neurological decline.^61^ These findings suggest that miR-191-5p not only reflects static injury burden but may also track with dynamic disease activity,

Taken together, the pronounced enrichment of miR-142-3p in nEVs and miR-191-5p in EVs shortly after PT, despite only minor changes in plasma, suggests that these miRNAs are involved in early CNS-specific signalling responses, possibly triggered by systemic inflammatory cues or stress-related neuronal activation. Their known roles in glutamatergic regulation, synaptic plasticity and neuroinflammation support the hypothesis that these vesicle-associated miRNAs may contribute to or reflect subclinical neuroinflammatory activity in trauma—even in the absence of overt brain injury.

### miR192-3p: early decrease in nEVs after TBI

miR192-3p was consistently detected in nEVs in healthy humans, suggesting a physiological role in maintaining CNS homoeostasis. Following trauma, miR-192-3p levels in nEVs showed a distinct temporal pattern: levels increased acutely after PT upon admission but declined after 48 h. In contrast, in TBI patients, miR-192-3p levels in nEVs increased at 48 h, indicating a dynamic regulation in response to systemic and neurological injury. Several studies implicate miR-192 in neuroprotection, neuroinflammation and cellular homoeostasis: In models of ischaemic stroke and cerebral injury, miR-192-5p was downregulated, and agomir-induced overexpression reduced infarct size, neuronal apoptosis and inflammatory cytokines, likely mediated through downregulation of tyrosine-phosphorylation-regulated kinase 1A (Dyrk1a), indicating a protective, anti-inflammatory function.^62^ In a murine model of chronic unpredictable mild stress-induced depression, miR-192-5p alleviated cognitive impairment via TGF-β1/Fbln2 signalling suppression, enhancing synaptic function and neurogenesis.^63^ In gouty arthritis and tendon fibrosis, miR-192-5p inhibited M1 macrophage polarization and reduced inflammatory cytokines, pointing to a role in modulating inflammatory responses.^64^ Interestingly, in models of peripheral nerve injury, inhibition of miR-192-5p has been shown to enhance axonal regeneration and reduce apoptosis, suggesting a beneficial role of its downregulation in peripheral nervous system repair.^65^ These findings indicate that miR-192-3p/5p exerts pleiotropic effects depending on the injury type, tissue context and timing. Notably, our data show that nEV-associated miR-192-3p is reduced in the acute phase after TBI and increases at 48 h, potentially reflecting an adaptive, delayed reparative response. The initial decrease could mirror neuronal stress or damage, while the subsequent increase may signal cell-intrinsic attempts at stabilization, such as modulation of apoptosis, suppression of neuroinflammation or promotion of synaptic remodelling.

### miR-338-3p: divergent kinetics in PT and TBI

miR338-3p exhibited distinct expression kinetics depending on injury type and time point. After PT, plasma and nEV levels increased early and declined after 48 h, correlating with thoracic injury severity, IL-10 levels and worse prognosis (RISC II). In line with this, we had previously observed upregulation of miR338-3p in the plasma of trauma patients. Its early expression may reflect lung injury or systemic inflammation, as supported by previous studies linking miR-338-3p to Activating Transcription Factor 4 (ATF4) signalling, resulting in aggravation sepsis-induced acute lung injury.^66,67^ ATF4 is activated in response to cellular stressors such as oxidative stress and hypoxia. While it initially promotes adaptive survival pathways, prolonged or unresolved stress conditions can lead to pro-apoptotic signalling.^68^ However, the mechanisms governing this functional shift remain incompletely elucidated. Moreover, both ATF4 and miR-338-3p have been implicated in bone biology. ATF4 regulates osteoblast differentiation and bone formation, while miR-338-3p influences osteogenic pathways and remodelling processes.^69,70^ Importantly, dysregulation of these pathways may contribute to pathological conditions. In the context of TBI, overactive ossification—such as heterotopic ossification—is a known complication, and emerging evidence suggests that aberrant ATF4 signalling may be involved.^71^

In contrast, miR338-3p levels in nEVs increased significantly 48 h after isolated TBI and correlated with injury severity (NISS), suggesting a time-dependent neurobiological response potentially linked to neuronal recovery or regulation of cellular stress. Previous studies highlight miR338-3p as a key regulator of neuronal development and polarity, with high expression in mature neurons and roles in dendritic architecture and tumour suppression.^72^ Its overexpression has also been associated with increased neuronal differentiation during cortical development,^73^ reinforcing its relevance for structural reorganization after injury. In the context of ischaemic brain injury, findings are more nuanced. miR-338-3p expression was downregulated following ischaemic stress in oxygen-glucose deprivation models. Its knockdown attenuated ischaemic injury and promoted neuronal survival, suggesting a role in modulating autophagy and neuroprotection.^74^ Further, a recent study by Feng *et al*.^75^ found elevated miR-338-3p levels associated with poor outcome in ischaemic stroke patients with high National Institutes of Health Stroke Scale scores, which is consistent with our observation of increased nEV-associated miR-338-3p levels correlating with TBI severity. This divergence between traumatic and ischaemic models may reflect different phases or mechanisms of injury response: whereas downregulation of miR-338-3p in ischaemia appears neuroprotective (e.g. by promoting autophagy), its late upregulation in TBI could indicate a transition towards regenerative processes. miR-338-3p also exerts anti-inflammatory and anti-apoptotic effects in non-neuronal tissues. In lipopolysaccharide-stimulated kidney epithelial cells, its overexpression reduced mitochondrial dysfunction, cytokine release and apoptosis, highlighting its broader role in cellular stress regulation and support its relevance in TBI-related secondary injury.^76^ Importantly, given its involvement in neuronal differentiation, myelination and axonal regeneration, our findings suggest that miR-338-3p may serve as a dynamic diagnostic and prognostic biomarker for TBI, reflecting both injury severity and the stage of neuroinflammatory or reparative processes. Modulating miR338-3p expression might offer a therapeutic strategy to enhance neurological recovery after TBI.

## Limitation

This study has several limitations. First, the relatively small sample size (*n* = 10 per group) limits the generalizability and statistical power of the findings. Second, among the various available isolation techniques, we selected size exclusion chromatography for EV isolation, as it has consistently provided sufficient yield and purity when working with small plasma volumes. While it is known that size exclusion chromatography-isolated EV fractions may contain residual plasma proteins, this is unlikely to significantly impact miRNA analysis. Although the compartment-specific analysis of plasma, EVs and neuron-derived EVs offers novel insights, the isolation and characterization of nEVs remain technically challenging. The use of L1CAM for neuronal enrichment, while widely applied, has been debated due to potential cross-reactivity and its expression in non-neuronal cells, which may affect the specificity of nEV isolation.

Another important limitation relates to the biological specificity of the selected miRNAs. Many of the investigated candidates, such as miR-21-5p, are known to be involved in a wide range of pathophysiological processes, including cancer, cardiovascular disease and systemic inflammation. Thus, while these miRNAs may reflect injury-associated biological responses, they cannot be considered injury-type-specific markers for TBI or PT. Their expression levels are influenced by both central and peripheral mechanisms, making it challenging to attribute observed changes to a particular organ system or pathology without complementary markers or functional assays. Additionally, the cellular origin of circulating and EV-associated miRNAs could not be fully resolved. While nEV enrichment increases CNS specificity, it does not definitively confirm neuronal origin without more refined cell-type-specific markers. Finally, although our miRNA panel was informed by previous sequencing and literature, broader profiling using high-throughput methods and functional validation in larger cohorts are needed to confirm the clinical utility of these markers.

## Conclusion

This study highlights the compartment-specific expression patterns of selected trauma- and neuroinflammation-associated miRNAs in plasma, EVs and nEVs following severe trauma. Among the miRNAs investigated, miR-338-3p showed the most distinct and compartment-specific expression pattern. While elevated in plasma and EVs during the early post-injury phase of PT—potentially reflecting systemic inflammation and tissue stress—miR-338-3p levels were markedly increased in neuro-derived EVs 48 h after isolated TBI. This delayed and CNS-specific upregulation may indicate its role in neuroregeneration or secondary injury mechanisms, underlining its promise for reflecting the extent of neuronal damage and ongoing pathophysiological processes. These findings emphasize the added value of compartmental miRNA analysis and suggest that longitudinal and compartment-aware profiling—especially of miR-338-3p—may improve the characterization and monitoring of post-TBI dynamics.

## Supplementary Material

fcaf242_Supplementary_Data

## Data Availability

Data are available on request to the responsible author.
