# Supplementary material for: MiR338-3p expression in extracellular vesicles after severe trauma with or without traumatic brain injury
Source: Brain Commun. 2025 Jun 21;7(4):fcaf242. doi: 10.1093/braincomms/fcaf242 (PMC12261298; doi:10.1093/braincomms/fcaf242)
Supplement: fcaf242_Supplementary_Data [file fcaf242_supplementary_data.docx]

**Supplementary material:** **Patients and Methods.**

Exclusion Criteria: Patients with known pre-existing immunological disorders, immunosuppressive medication, burns, concomitant acute myocardial infarction, thromboembolic events, or death within two days of hospital admission (resulting in incomplete serial blood sampling) were excluded from the study.

Sampling Time Points: Available data from preliminary studies suggest that neuronal and inflammatory biomarkers are expressed in a time-dependent manner after trauma. In the case of primary brain injury, the early post-injury phases a particularly critical. In addition, secondary brain injury - driven by neuroinflammatory processes - typically evolves within 6 to 48 hours after trauma. Therefore, patients' blood samples were collected at two defined time points: within ≤3 hours of emergency room admission (referred to as “ER”) and at 48 hours post-injury.

Micro(mi)RNA and Extracellular Vesicle (EV) isolation: The blood was collected in EDTA-K tubes and centrifuged at 3500 rpm for 15 minutes at 4 °C. The upper plasma phase was carefully separated and stored at -80 °C until further analysis. Extracellular vesicles were isolated from 100 µl of plasma using size exclusion chromatography (Exo-Spin TM, EV size exclusion column, cell guidance systems, Cambridge, UK) according to the manufacturer's instructions. Number and size distribution of EV particles were determined by nanoparticle tracking analysis (NTA) (Nanosight NS500, Malvern Panalytical, Kassel, Germany). For the enrichment of neuro-specific (n) EVs, magnetic beads (Exosome-Streptavidin Isolation/Detection Reagent; Thermofisher Scientific) were conjugated to a biotinylated anti-L1CAM antibody (eBio5G3 (5G3)), Biotin, eBioscience™) following the manufacturer's protocol. Plasma-derived EVs were incubated with the prepared beads, and bead-bound nEVs were isolated using a magnetic separator and used directly for miRNA isolation.

MiRNA was isolated from 100 µl of plasma, EVs, or nEVs (each derived from 100 µl of plasma) using the miRNeasy serum/plasma Kit (Qiagen Inc., Hilden, Germany) according to the manufacturer's instructions. Reverse transcription was performed using an amount of RBA equivalent to that extracted from 50 µl of plasma, with the miRCURY LNA RT Kit (Qiagen Inc., Hilden, Germany), including the spike-in cel-miR-39 control (miRNA spike-In Kit, for RT, Qiagen Inc., Germany). Quantitative PCR was using the miScript SYBR® Green PCR Kit (Qiagen Inc., Hilden, Germany) on a CFX96 Touch Real-Time PCR Detection System (BioRad, Puchheim, Germany). Cycling conditions included an initial denaturation step at 95 °C for 3 minutes, followed by 40 cycles of 95 °C for 10 seconds, and 56 °C for 50 seconds, ending with melting curve analysis. The exogenous spike-in control cel-miR-39 was measured to account for differences in RNA extraction efficiency and reverse transcription.
